# Viral Mimicking Polyplexes as Hierarchical Unpacking Vectors for Rheumatoid Arthritis Treatment

**DOI:** 10.1002/advs.202402888

**Published:** 2024-06-25

**Authors:** Haofang Zhu, Danqing Huang, Jinglin Wang, Yuanjin Zhao, Lingyun Sun

**Affiliations:** ^1^ Department of Rheumatology and Immunology The First Affiliated Hospital of Anhui Medical University 218 Jixi Road Hefei 230022 P.R. China; ^2^ Department of Rheumatology and Immunology Institute of Translational Medicine The Affiliated Drum Tower Hospital of Nanjing University Medical School 321 Zhongshan Road Nanjing 210008 P. R. China; ^3^ State Key Laboratory of Bioelectronics School of Biological Science and Medical Engineering Southeast University 2 Sipailou Nanjing 210096 P. R. China

**Keywords:** cell‐free DNA, cyclosporin A, rheumatoid arthritis, ternary polyplexes, viral‐mimicking

## Abstract

Nano‐delivery systems hold great promise for the treatment of rheumatoid arthritis (RA). Current research efforts are primarily focused on enhancing their targeting capabilities and efficacy. Here, this study proposes a novel viral‐mimicking ternary polyplexes system for the controlled delivery of the anti‐inflammatory drug Cyclosporin A (CsA) to effectively treat RA. The ternary polyplexes consist of a nanogel core loaded with CsA and a hyaluronic acid shell, which facilitates CD44‐mediated targeting. By mimicking the Trojan Horse strategy employed by viruses, these polyplexes undergo a stepwise process of deshielding and disintegration within the inflamed joints. This process leads to the release of CsA within the cells and the scavenging of pathogenic factors. This study demonstrates that these viral‐mimicking ternary polyplexes exhibit rapid targeting, high accumulation, and prolonged persistence in the joints of RA mice. As a result, they effectively reduce inflammation and alleviate symptoms. These results highlight the potential of viral‐mimicking ternary polyplexes as a promising therapeutic approach for the targeted and programmed delivery of drugs to treat not only RA but also other autoimmune diseases.

## Graphical abstract

Nano‐delivery systems offer a new horizon for rheumatoid arthritis (RA) treatment. The innovative viral‐mimicking ternary polyplexes system promises targeted delivery of Cyclosporin A, using Trojan Horse‐like mechanism to ensure direct drug release in inflamed joints, neutralizing harmful factors. Proven in RA mice, this method shows significant inflammation reduction and symptom relief, marking a step forward in autoimmune disease therapy.

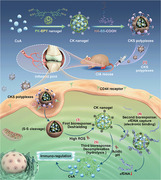

## 1 Introduction

Rheumatoid arthritis (RA) is a chronic, autoimmune, and potentially devastating disorder. In the pathogenesis of RA, proinflammatory cytokines and damage‐associated cell‐free DNAs (cfDNAs) play critical roles.^[^ ^1^, ^2^ ^]^ These excessive factors trigger and sustain the inflammatory response in immune cells, leading to joint damage and pain.^[^ ^3^, ^4^ ^]^ Clinically, Cyclosporin A (CsA) is recognized for its efficacy as a Disease Modifying Anti‐Rheumatic Agent (DMARD), particularly in cases where other treatments have proven ineffective.^[^ ^5^, ^6^ ^]^ Through systematic administration, CsA can relieve symptoms due to its anti‐inflammatory and immunoregulatory properties.^[^ ^7^, ^8^ ^]^ Whereas, the direct administration of CsA is often complicated by drug resistance, fast metabolism, and a broad range of adverse effects such as infections. Recent decades, with the robust development of nanotechnology, various nanocarriers have been widely applied in medications.^[^ ^9^, ^10^, ^11^, ^12^, ^13^, ^14^, ^15^, ^16^ ^]^ Although some reported nano‐delivery systems have shown prolonged circulation time, their biocompatibility and targeting capacity are still far from satisfied. Therefore, smart nano‐systems that can precisely deliver and controllably release drugs are still anticipated for improving the anti‐RA efficacy.

**Figure 1:**
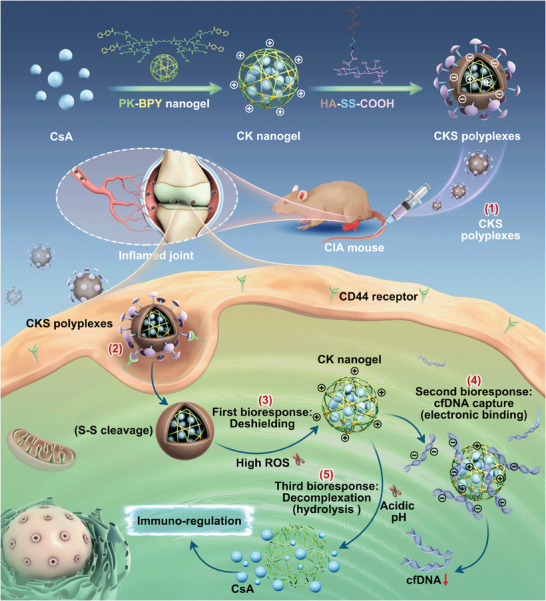
Schematic representation of CKS ternary polyplexes for RA treatment. 1) ternary polyplex formation by introduction of HA‐SS‐COOH to CsA/PK‐BPY (CK) nanogel; 2) HA‐receptor‐mediated endocytosis; 3) Reactive oxygen species (ROS)‐triggered deshielding of HA‐SS‐COOH; 4) cfDNA capture by CK nanogel via electronic interaction; 5) hydrolysis of CK nanogel and CsA release.

Here, inspired by the sophisticated structures and functions of virus, we proposed a novel nanocarrier with viral‐mimicking core‐shell structures for programmed and targeting CsA delivery to treat RA, as schemed in **Figure** **1**. Viruses are powerful natural masterpieces with a well‐organized nanoscale core‐shell structure.^[^ ^17^, ^18^, ^19^, ^20^ ^]^ Interestingly, the targeting ability of the shell and the stimulus responsiveness of the core endow viruses with the capacity for targeted gene delivery and hierarchical gene release.^[^ ^21^, ^22^, ^23^ ^]^ By employing a clever Trojan Horse strategy, they invade host cells and progressively unpack the envelope to release their payloads.^[^ ^24^, ^25^, ^26^, ^27^ ^]^ Based on the distinctive properties of the virus's special structure, some ongoing trials have developed virus‐inspired nanomaterials to deliver medicines and have shown promising results.^[^ ^28^, ^29^, ^30^, ^31^ ^]^ However, the undesired biocompatibility, bio‐adaptability, intelligence in their composition, and adverse immune responses limited their further application.^[^ ^32^, ^33^ ^]^

In this paper, we employed hierarchically unpacking ternary polyplexes as the virus‐like nanocarriers for programmed CsA delivery and targeting RA treatment. By using polyethylene glycol (PEG)‐conjugated poly‐lysine (K) dendrimer (PK), a nanogel core encapsulating CsA can be formed. Subsequently, the core can be further shielded by disulfide‐modified hyaluronic acid (HA) derivatives (HA‐SS‐COOH), bringing about the construction of ternary polyplexes (CsA/PK/HA‐SS‐COOH, CKS). Like the Trojan Horse strategy, the CKS polyplexes could protect the inner nanogel core under general physiological conditions to enhance cellular internalization by CD44 recognition.^[^ ^34^, ^35^, ^36^ ^]^ Besides, the ternary polyplexes can undergo first‐stage unpacking during cellular attachment or just after cellular uptake, followed by continuously unpacking for CsA release. In contrast, the cationic nanogel core could efficiently scavenge the intracellular cfDNA and dampen inflammation. It was found that after intravenously administration, the CKS polyplexes displayed preferential accumulation and prolonged retention in the inflamed joints of RA mice, thereby effectively alleviating disease symptoms. These results elucidated that our viral‐mimicking nanostructures imparted the ternary polyplexes with targeted and programmed CsA delivery ability, showing potential utility in treating RA and other autoimmune disorders.

## 2 Results and Discussion

### 2.1 Synthesis and Characterization of CKS Polyplexes

The preparation of PK‐2,2′‐bipyridine‐4‐carboxylic acid (BPY) polymers and HA‐SS‐COOH are described in Figures S1 and S2 (Supporting Information). The successful synthesis of PK‐BPY and HA‐SS‐COOH was verified using the Matrix‐assisted laser desorption/ionization time‐of‐flight mass spectrum (MALDI‐TOF‐MS, Figure S3, Supporting Information) and ^1^H NMR spectrum (Figure S4, Supporting Information). The PK‐BPY nanogels were formed through aromatic interactions between BPY in aqueous solution, and CsA was loaded into the nanogel core to create CsA/PK nanogel (CK). The drug loading content (DLC) and drug loading efficiency (DLE) of CsA were determined as 20.5% and 93.5%. Then, the ternary polyplexes (CKS) were obtained by coating the cationic CK nanogels with the anionic HA‐SS‐COOH through electronic interaction.

**Figure 2:**
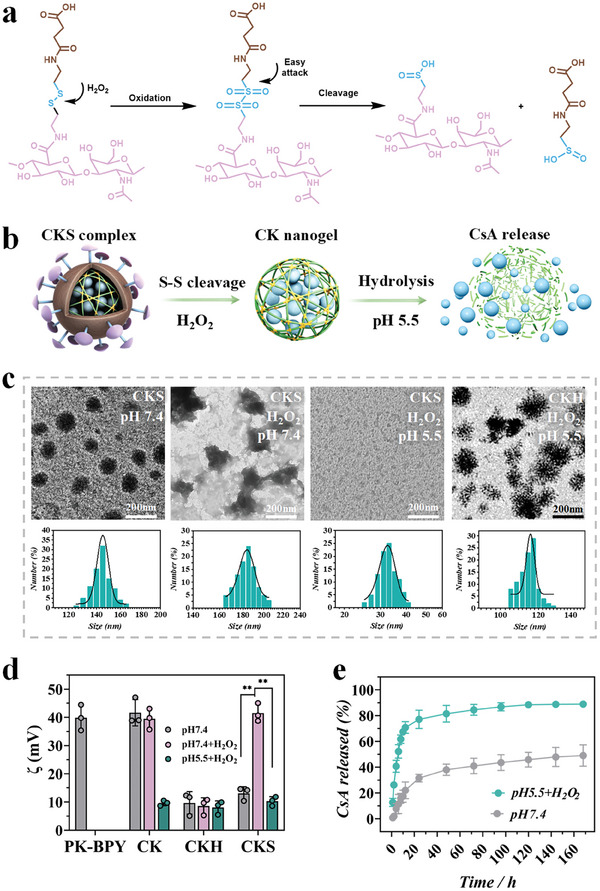
The characterization of CKS polyplexes. a) The oxidation‐triggered deshielding of disulfide bonds and the subsequent hydrolysis of CK nanogel and CsA release. b) The stepwise deshielding and unpacking of CKS polyplexes in response to inflammatory microenvironment. c) Oxidation and acidic pH responses of morphology and size distributions of polyplexes. Polyplexes were exposed to different buffer conditions for 1 h. Size distributions were recorded by intensity. d) Zeta‐potential variation of different polyplexes at indicated microenvironments (pH 7.4 without H_2_O_2_, pH 7.4 with 1 mm H_2_O_2_, and pH 5.5 with 1 mm H_2_O_2_). e) CsA release profile at pH 5.5 with 1 mm H_2_O_2_ or at pH 7.4 without H_2_O_2_). Data were presented as mean ± SD. Statistical significance was calculated by one‐way ANOVA followed by post hoc tests, *0.01 < *P* < 0.05, **0.001 < *P* < 0.01, ***P < 0.001.

The design of these polyplexes aims to achieve “deshielding” by degrading the outer HA‐SS‐COOH shell through breaking disulfide bonds in the presence of high concentrations of reactive oxygen species (ROS) (**Figure** **2**a,b), which was at millimolar (1–10 mm) levels in the intracellular compartments of inflammatory cells in RA joints.^[^ ^37^, ^38^ ^]^ Transmission Electron Microscope (TEM) scanning and dynamic light scattering (DLS) confirmed the spherical morphologies of CKS and CKH (CsA/PK/HA without disulfide bonds) polyplexes (Figure 2b). Once the CKS been endocytosed, intracellular ROS could destroy the disulfide and hasten the dissolution of outer HA shell. The TEM images showed a similar uniform spherical morphology of CKS and CKH polyplexes with diameters of ≈130 nm. The size of the CKS polyplexes increased upon exposure to1 mm H_2_O_2_, indicating the swelling of the loose part around the core due to disulfide cleavage in HA‐SS‐COOH. Treatment with a a lower pH 5.5 resulted in the complete cleavage of disulfide bonds and hydrolysis of the CK nanogel core, leading to smaller and irregularly shaped polyplexes.

To investigate the first‐stage deshielding and second‐stage unpacking of CKS polyplexes, we measured the zeta potential of the polyplexes at different ROS concentrations and pH (pH 7.4 without H_2_O_2_, pH 7.4 with 1 mm H_2_O_2_, and pH 5.5 with 1 mm H_2_O_2_) (Figure 2d). The pH 7.4 condition without H_2_O_2_ and pH 5.5 condition with 1 mm H_2_O_2_ correspond to the physiological environment and inflammatory intracellular condition in RA joints, respectively. In the absence of H_2_O_2_, the zeta potential of CKS was +13.1 mV, which was significantly lower than that of CK nanogels (+41.6 mV). Treatment with 1 mm H_2_O_2_ caused a sharp increase in the zeta potential of CKS to +40.7 mV. As the pH was further decreased to 5.5, the zeta potential decreased to +10.5 mV, which was similar to the zeta potential of the CK nanogels (+8.7 mV). In contrast, the nonresponsive shielded CKH did not show any increase in zeta potential at 1 mm H_2_O_2_. These results indicate that the HA‐SS‐COOH deshielding only occurred in CKS polyplexes under inflammatory conditions, leading to partial exposure of the positively charged CK core and an increase in positive zeta potential. The inner CK core underwent decompaction through hydrolysis at pH 5.5, resulting in the release of CsA and small pieces of PK with a decreased zeta potential. Overall, these findings demonstrate the stepwise deshielding and unpacking of CKS polyplexes in response to inflammatory microenvironment, highlighting their potential for programmable drug delivery in inflamed joints.

Next, we evaluated the drug release behavior of CKS based on this hierarchical unpacking strategy. The drug release profiles of CsA from CKS at pH 7.4 without H_2_O_2_ and at pH 5.5 with 1 mm H_2_O_2_ were evaluated. It was found that CsA was slowly and constantly released from the nanogels at both conditions (Figure 2e). However, a higher release rate was observed in the presence of H_2_O_2_ and lower pH, indicating the desired intracellular degradation of CKS triggered by ROS and acidic pH.

Prior to biological evaluation, the cytocompatibility of the polyplexes was assessed using mouse macrophages (RAW 264.7 cells), and negligible cytotoxicity was observed at a concentration of 500 µg mL^−1^ (Figure S5, Supporting Information), which was attributed to its nature‐derived peptide components and protein‐like globular structure. This characterization of CKS polyplexes and their controlled drug release behavior provides important insights into the potential of these viral‐mimicking ternary polyplexes for programmable drug delivery in the treatment of rheumatoid arthritis.

### 2.2 CKS Polyplexes Shows High Cellular Uptaking Efficiency and Superior DNA Binding Efficiency

**Figure 3:**
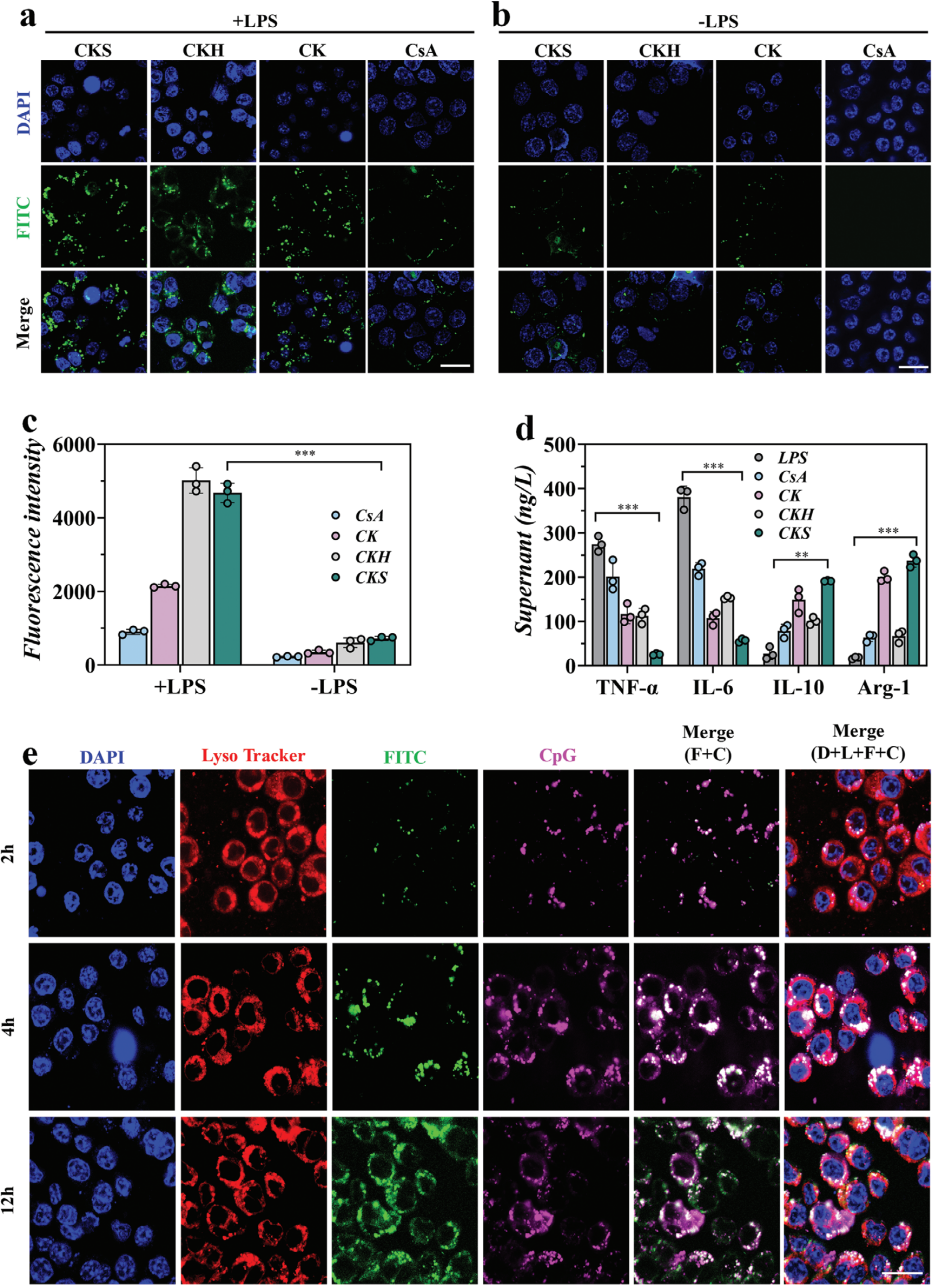
The cellular uptake and binding efficiency of CKS polyplexes with intracellular cfDNA. Fluorescent images of a) activated RAW 264.7 cells (by LPS) and b) inactivated RAW 264.7 cells treating with FITC‐labelled CKS, CKH, CK or CsA for 2 h. Scale bar = 100 µm. c) Quantified fluorescence intensity from a) and b). d) Supernatant cytokines were detected in RAW 264.7 cells treated by LPS, CsA, CK, CKH, and CKS. e) Intracellular trafficking of Cy5.5‐labeled CpG and FITC‐labeled cationic CKS nanogels in RAW264.7 cells after at different time points. Scale bar = 100 µm. White spots marked by the white arrows indicating the colocalization of CpG and CKS nanogels. D: DAPI, L: Lysosome Tracker, F: FITC‐labeled CKS, and C: Cy5.5‐labeled CpG. Data were presented as mean ± SD. Statistical significance was calculated by one‐way ANOVA followed by post hoc tests, *0.01 < *P* < 0.05, **0.001 < *P* < 0.01, ***P < 0.001.

To further evaluate the efficacy of the prepared polyplexes, we conducted a macrophage uptake assay. Macrophages play a crucial role in the secretion of inflammatory mediators, such as Tumor Necrosis Factor‐*α* (TNF‐*α*), Interleukin‐6 (IL‐6), and Interleukin‐1*β* (IL‐1*β*), and are abundantly present at inflammatory sites. Therefore, assessing the uptake of the polyplexes by activated macrophages is essential to determine the efficiency of the formulation for treating rheumatoid arthritis (RA). Both non‐activated and lipopolysaccharide (LPS)‐activated macrophages, which express the HA receptor CD44, were used in the macrophage uptake assay. **Figure** **3**a clearly shows strong green fluorescence throughout the activated macrophages, indicating substantial cellular uptake mediated by HA‐CD44 recognition. The uptake of free CsA by non‐activated and activated macrophages was similar, but only weak green fluorescence was observed in a portion of the cells compared to non‐activated macrophages (Figure 3b).

To have a quantitative characterization, the fluorescent intensities were quantified (Figure 3c). The activated macrophages exhibited approximately fivefold higher fluorescence intensity with CKS/CKH compared to normal macrophages. In contrast, the fluorescence intensity of CK nanogel was low in both activated and normal macrophages. It is worth noting that normal macrophages exhibited minimal uptake for all samples, demonstrating the inflammation targeting ability of the CKS polyplexes.

To further validate the ability of CKS polyplexes to reprogram macrophages, we measured the expression levels of cytokines associated with proinflammatory and anti‐inflammatory responses, including TNF‐*α*, IL‐6, Arg‐1, and IL‐10, using ELISA. Macrophages cocultured with CKS polyplexes exhibited a significant decrease in the expression levels of proinflammatory TNF‐*α* and IL‐6, while showing increased expression of anti‐inflammatory Arg‐1 and IL‐10 compared to the control group (Figure 3d). These findings collectively demonstrate the anti‐inflammatory activity of CKS polyplexes by promoting the differentiation of macrophages into the anti‐inflammatory M2 subtype.

To investigate the mechanisms underlying cellular uptake and macrophage reprogramming in inflammation suppression, we used confocal laser scanning microscopy (CLSM) to observe the extra‐ and intracellular trafficking of Cy5.5‐labeled CpG‐ODN2006 and FITC‐labeled polyplexes (Figure S6, Supporting Information) in RAW 264.7 cells. The cells were co‐incubated with the labeled cfDNA and polyplexes for 12 h. Remarkably, the incorporation of CKS polyplexes led to a significant reduction in the internalized cfDNAs (Figure 3e). This observation suggests that the exposed cationic CK nanogels can recognize, scavenge, and inhibit the internalization of inflammatory cfDNA through electronic interactions.

Previous studies have shown that anionic cfDNA activates immune cells and induces the production of proinflammatory cytokines through the TLR‐MyD88‐NFκB signaling pathway.^[^ ^39^ ^]^ The exposed cationic CK nanogels, with their positive surface charge, can bind and scavenge anionic cfDNA through electronic interactions. This binding inhibits the intracellular trafficking of cfDNA from the plasma membrane to the endolysosomes, preventing it from reaching DNA sensors, such as TLR9, located on the endolysosomes. By clearing cfDNA, the ligand for TLR9 is reduced, resulting in decreased activation of TLR9 and its adaptor molecules, MyD88 and TRAF6. Consequently, there is less phosphorylation and degradation of IκB*α*, an inhibitor of NFκB. As a result, NFκB is unable to translocate to the nucleus and induce the expression of inflammatory genes. These findings suggest that the CKS polyplexes are capable of effectively clearing inflammatory cfDNA and modulating the TLR‐MyD88‐NFκB signaling pathway, thereby suppressing inflammation. This mechanism highlights the potential of CKS polyplexes for the treatment of inflammatory diseases.

### 2.3 CKS Inhibits the Migration and Invasion of RAFLS

**Figure 4:**
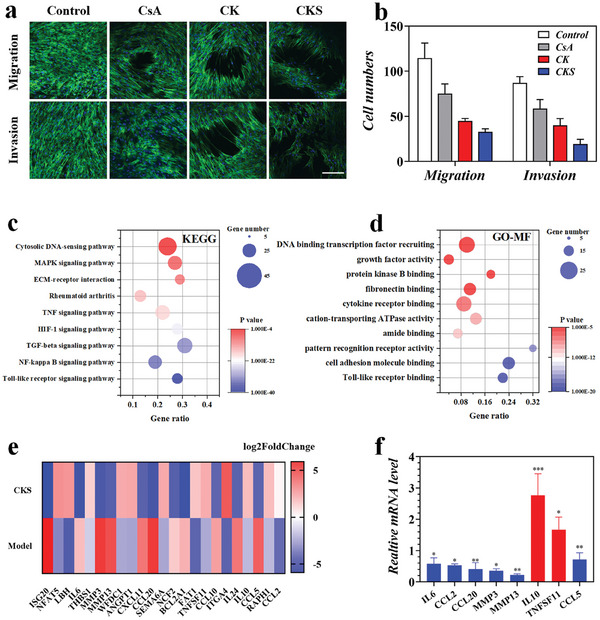
CKS inhibited RAFLS migration, invasion, and inflammatory responses in vitro. a) Actin/diamidino phenylindole (DAPI) staining of RAFLS in migration and invasion assays. The scale bar is 200 µm. b) Quantification of RAFLS cells migrated and invaded through Trans well. c) KEGG and d) GO pathway analyses of the target genes of the top ten significantly expressed miRNAs in the CKS group compared with the NC group. The GO terms and KEGG pathway terms enriched in the predicted target genes of the miRNAs were analyzed using Database for Annotation Visualization and Integrated Discovery (DAVID) Bioinformatics. MF, molecular functions. e) The expression of marker genes in CKS group and NC group. f) Expression analysis of the selected genes by qPCR. Data were presented as mean ± SD. Statistical significance was calculated by one‐way ANOVA followed by post hoc tests, *0.01 < *P* < 0.05, **0.001 < *P* < 0.01, ***P < 0.001.

To investigate the potential inhibitory effects of CKS polyplexes on the migration and invasion of RA fibroblast‐like synoviocytes (RAFLS), transwell experiments were conducted by incubating RAFLS with CsA, CK, and CKS polyplexes. The results demonstrated that both CK and CKS polyplexes effectively inhibited the migration and invasion of RAFLS. Interestingly, CKS polyplexes exhibited superior inhibitory effects on RAFLS compared to CK alone (**Figure** **4**a,b), suggesting that the viral‐mimicking ternary structure plays a critical role in regulating FLS function and RA progression.

To elucidate the mechanisms underlying the inhibitory effects of CKS polyplexes on FLS migration and invasion, RNA sequencing (RNA‐seq) experiments were performed on FLS incubated with CKS polyplexes (CKS group) and Matrigel (NC group). Comparative analysis revealed the differential upregulation of 3595 genes and downregulation of 3148 genes in the CKS group compared to the NC group (*p* < 0.001) (Figure S7, Supporting Information). KEGG analysis detected the enrichment of nine signaling pathways highly associated with FLS function in RA development, including Rheumatoid arthritis, INF signaling pathway, HIF‐1 signaling pathway, TGF beta signaling pathway, NF‐kappa B signaling pathway, and Toll‐like receptor signaling pathway (Figure 4c). GO enrichment analysis revealed that the effects of CKS on FLS were related to various molecular functions, biological processes, and cellular components, such as “Toll‐like receptor binding,” “cell adhesion molecule binding,” “negative regulation of cell migration,” “extracellular matrix organization,” “protein‐DNA complex,” and “nuclear euchromatin” (Figure 4d; Figure S8, Supporting Information). Heatmap analysis identified the top 23 marker genes with significantly different expression in the highly enriched pathways, including chemokine C‐C motif Ligand (CCL)‐5, IL6, Matrix Metalloproteinases (MMP)‐13, MMP3, and Tumor Necrosis Factor Superfamily (TNFSF)‐11, which were crucial genes involved in the downregulation of FLS migration (Figure 4e). The expression levels of these genes were further validated by quantitative real‐time polymerase chain reaction (qPCR), and the results were consistent with the RNA‐seq analysis (Figure 4f).

These findings provide insights into the molecular mechanisms underlying the inhibitory effects of CKS polyplexes on FLS migration and invasion, highlighting the potential of CKS polyplexes as a therapeutic strategy for RA.

### 2.4 CKS Achieves an Extremely Fast Targeting Accumulation in RA Joints

**Figure 5:**
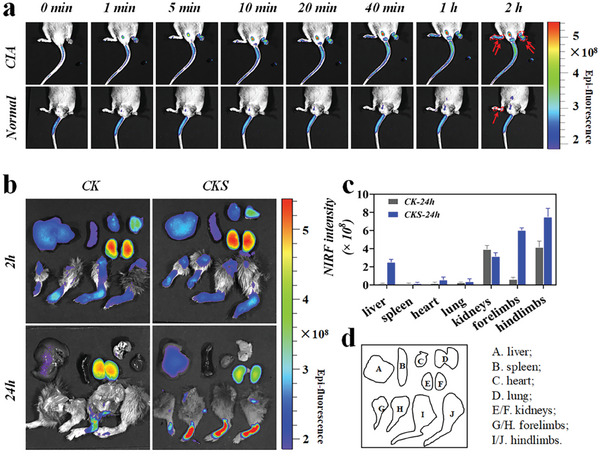
The mechanistic studies of the favorable therapeutic performance of CKS polyplexes in treating RA. a) Dynamic in vivo NIRF images of CKS from 0 to 2 h. b) The biodistribution of CKS in extra‐articular organs and joints at 2 and 24 h post‐injection by ex vivo NIRF imaging. Quantification of mean NIRF intensity of CKS in ex vivo biodistribution study of CIA mice at c) 2 h and d) 24 h after i.v. injection. e) A). liver; B). spleen; C). heart; D). lung; E/F). kidneys; G/H). forelimbs; I/J). hindlimbs. Data are expressed as mean ± SD.

To assess the in vivo targeting efficiency of CKS polyplexes, Near‐infrared fluorescence (NIRF) imaging was performed. Alexa Flour 750 (AF750)‐labeled CKS (Figure S9, Supporting Information) were prepared and injected intravenously into collagen‐induced arthritis (CIA) mice. NIRF imaging revealed rapid movement of CKS polyplexes through the tail vein and immediate accumulation in the inflamed joints of CIA mice within minutes of injection (**Figure** **5**a). After 2 h, a strong NIR signal from AF750‐labeled CKS was observed in the inflamed joints of CIA mice, while negligible fluorescence was detected in the joints of normal mice. Movie files illustrating the path of CKS post‐injection in both CIA and normal mice were also available (Movies S1 and S2, Supporting Information, respectively).

The biodistribution of CKS in extra‐articular organs and joints was measured (Figure 5b). At 2 h post‐injection, a higher accumulation of CKS polyplexes was observed in the joints of CIA mice compared to CK nanogel. This suggests that the outer HA shell of CKS is crucial for specific targeting to inflamed cell membranes. After 24 h, CKS polyplexes still exhibited significant accumulation in the joints and paws, indicating that the hierarchical structure of CKS prolongs joint retention time (Figure 5c).

### 2.5 CKS Relieves Symptoms of CIA Mice in Therapeutic Treatment

**Figure 6:**
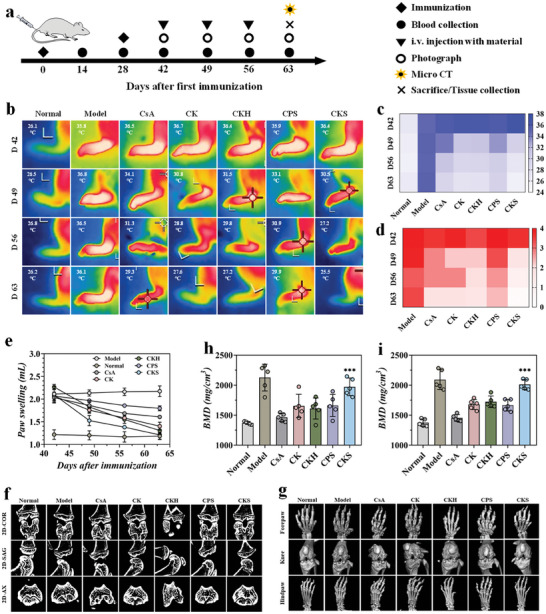
In vivo therapeutic effects of CKS polyplexes in the CIA model. a) Schematic representation of the establishment and treatment of the CIA mice model. B) Thermographic images of left hind paws and corresponding quantification of c) paw temperatures, d) clinical scores, and e) paw swelling at various time points after treatment. f) Representative 2D reconstructed micro‐CT images in the COR, SAG, and AX planes of the knee joints. g) Representative 3D reconstructed micro‐CT images of the knee and paw joints. The BMD of the h) ankle and i) knee joints of mice in different treatment groups. n = 5, biologically independent samples. Data were presented as mean ± s.e.m. Statistical significance was calculated by one‐way ANOVA followed by post hoc tests, *0.01 < *P* < 0.05, **0.001 < *P* < 0.01, ***P < 0.001.

The therapeutic performance of CKS polyplexes was evaluated in a mouse CIA model that mimics the pathological features of human RA. Starting from day 42, CKS polyplexes were administered intravenously at a dose of 25 mg kg^−1^ of body weight weekly for four weeks (**Figure** **6**a). From day 49 onwards, all cationic nanogels showed reduced swelling and erythema in hind paws, with the CKS polyplexes group showing the most significant improvement. Treatment with CKS resulted in a near‐normal level of hindpaw swelling by day 70. Moreover, both hindpaws and forepaws of CKS‐treated mice exhibited significantly improved clinical scores, indicating the positive therapeutic effects of the programmed drug delivery (Figure 6b−e).

Bone erosion in CIA mice was monitored using micro‐CT imaging (Figure 6f,g). All treatment groups showed varying levels of bone destruction in the ankle and knee joints. Bone mineral density (BMD) was calculated from micro‐CT reconstructions (Figure 6h,i). The CIA model group exhibited a significant decrease in BMD for ankle and knee joints by day 29, indicating severe bone destruction. Treatment with CsA, CK, CKH, and CsA/commercial PAMAM dendrimer/HA‐SS‐COOH (CPS) polyplexes resulted in a slight improvement in BMD between days 42 and 70. In contrast, CKS polyplexes treatment restored BMD levels to normal, indicating evident inhibition of erosive bone damage.

To evaluate the importance of ROS‐responsive degradation of the HA‐SS‐COOH shell, which is crucial for the in vivo degradation of CKS in response to the inflammatory microenvironment, we employed HPLC to examine the pharmacokinetics of CKS and CKH complexes after intravenous injection into mice, respectively. The mean plasma concentrations after intravenous administration of a single 10 mg kg^−1^ dose of CKS and CKH in mice are depicted in Figure S10 (Supporting Information). The plasma concentration of CsA was graphed against time to depict its in vivo pharmacokinetic behavior. The graph unveiled a steady increase and consistent plasma levels of CsA, reaching a C_max_ of 153.15 ± 9.52 ng mL^−1^ at T_max_ of 48 h, signifying an extended blood circulation time for CKS. In contrast, CKH exhibited a C_max_ of 45.22 ± 3.68 ng mL^−1^ at T_max_ of 36 h, indicating a relatively slow drug release and low drug availability attributed to its non‐responsive HA shell.

**Figure 7:**
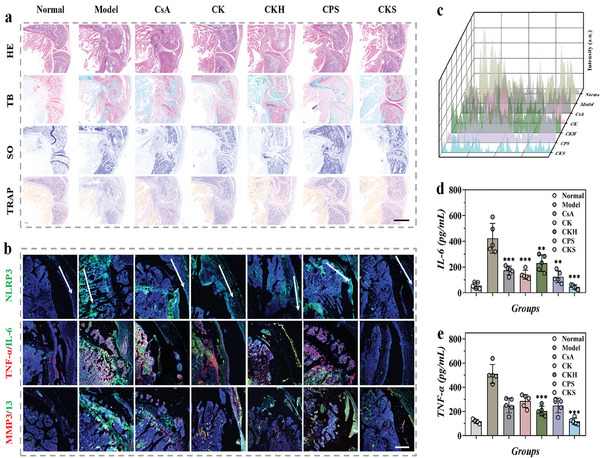
In vivo histology outcomes of RA with the treatment of CKS polyplexes. a) H&E, toluidine blue, safranin O, and TRAP staining, and b) NLRP‐3, TNF‐*α*, IL‐6, MMP3/13 immunofluorescent staining of the paw joint of CIA mice at day 63 after immunization. c) Quantification of NLRP‐3 expression were confirmed by line profile analysis. Scale bar, 200 µm. Serum cytokines of d) IL‐6 and e) TNF‐*α* were detected at day 63 after immunization. Data were presented as mean ± s.e.m. Statistical significance was calculated by one‐way ANOVA followed by post hoc tests, *0.01 < *P* < 0.05, **0.001 < *P* < 0.01, ***P < 0.001.

Histological analysis of RA joints using Hematoxylin‐Eosin (H&E) staining, toluidine blue (TB), safranin O (SO), and tartrate‐resistant acid phosphatase (TRAP) staining revealed severe bone and synovium destruction, decreased chondrocytes, and intense leukocyte invasion in the model group (**Figure** **7**a). In contrast, CKS‐treated mice showed considerable restoration of soft tissues. Staining with toluidine blue and safranin O indicated apparent cartilage loss in the model group, indicating severe joint damage. However, CKS treatment led to a restoration of cartilage integrity. The number of TRAP‐positive cells, indicative of osteoclasts, decreased with CKS treatment. Additionally, the CKS group exhibited a nearly normal synovial boundary, similar to the healthy group, suggesting a potential cartilage protective effect of CKS polyplexes in RA treatment.

Immunofluorescent staining of paw joints further supported the therapeutic effects of CKS polyplexes (Figure 7b). Key diagnostic biomarkers related to the pattern recognition receptor (PRR) signaling pathways of RA, including TNF‐*α*, IL‐6, and collagen‐degrading MMP‐3/13, were measured. CKS treatment significantly reduced the expression of inflammatory cytokines and MMP‐3/13, bringing them down to normal levels (Figures S11–S13, Supporting Information). In comparison, the CsA, CK, CKH, and CPS treated groups showed only a certain degree of reduction in inflammation and collagen degradation (Figure 7c). These findings were further supported by the analysis of systemic cytokine levels, where CKS treatment restored TNF‐*α* and IL‐6 expression to levels comparable to normal mice, indicating a more effective inhibition of RA (Figure 7d,e). These anti‐inflammatory mechanisms demonstrate the superior therapeutic efficacy of CKS polyplexes in RA treatment.

The potential toxicity of cationic biomaterials is a major concern for clinical translation. However, CKS polyplexes exhibited favorable therapeutic performance in ameliorating RA progression while displaying minimal toxicity to major organs (Figure S14, Supporting Information), consistent with in vitro studies. The favorable biocompatibility of CKS polyplexes may be attributed to their naturally derived peptide and polysaccharide components, as well as their specific targeting and biodistribution profile in inflamed joints. This allows for localized and programmed delivery of CsA to relieve RA progression without systemic side effects.

## 3 Conclusion

In this paper, we developed a novel viral‐mimicking ternary polyplexes system that can deliver CsA to the inflamed joints of RA mice in a programmed manner. By mimicking the virus's Trojan Horse strategy, our system can overcome the challenges of poor bioavailability, low targeting efficiency, and systemic toxicity of CsA. We have demonstrated that our system can achieve fast and selective targeting, high and sustained accumulation, and efficient and controlled release of CsA in the joints of RA mice. As a result, our system can effectively reduce the inflammation and relieve the symptoms of RA in a mouse model. Our findings suggest that our viral‐mimicking ternary polyplexes system is a promising candidate for treating RA and other autoimmune diseases that require local and long‐term anti‐inflammatory therapy.

## Conflict of Interest

The authors declare no conflict of interest.

## Supporting information

Supporting Information

Supplemental Movie 1

Supplemental Movie 2

## Data Availability

The data that support the findings of this study are available in the supplementary material of this article.
