# Supplementary material for: Viral Mimicking Polyplexes as Hierarchical Unpacking Vectors for Rheumatoid Arthritis Treatment
Source: Adv Sci (Weinh). 2024 Jun 25;11(32):2402888. doi: 10.1002/advs.202402888 (PMC11348054; doi:10.1002/advs.202402888)
Supplement: Supplementary file 1 — Supporting Information [file ADVS-11-2402888-s002.docx]

Supporting Information

Viral mimicking polyplexes as hierarchical unpacking vectors for rheumatoid arthritis treatment

*Haofang Zhu^a,b^, Danqing Huang^a^, Jinglin Wang^a^, Yuanjin Zhao^a,c,^*, Lingyun Sun^a,b^**

**Materials and Methods**

*Materials*: H-Lys-OMe 2HCl, Boc-Lys(Boc)-OH, 1-hydroxybenzotriazole hydrate (HOBT), N-(3-dimethylaminopropyl)-N’-ethylcarbodiimide hydrochloride (EDCI), N, N-diisopropylethylamine (DIPEA) and TFA were commercial available from GL Biochem Ltd (Shanghai, China). NH2-PEG2000-NH2 was purchased from Shanghai Ponsure Biotech, Inc. Regenerated Cellulose Dialysis Membrane (MWCO = 10 kDa) were obtained from Spectrum/Por (Houston, USA). All other chemicals were of analytical grade. CCK-8 assay kit, Calcein AM, and propidium iodide (PI) were commercially available from KeyGEN BioTECH Co., Ltd (Nanjing, China). Fluorescein isothiocyanate (FITC, Aladdin, China) was used in its stock solution, 10 mg/mL, in DMSO. Alexa Fluor® 750 NHS Ester (succinimidyl ester) (AF750-NHS, Invitrogen) was dissolved in DMSO to prepare a stock solution of 10 mg/mL. Phosphate buffered solution (PBS, pH = 7.4), Dulbecco’s modified Eagle medium (DMEM), Iscove’s modified Dulbecco’s media (IMDM) and fetal bovine serum (FBS) were purchased from United States Origin, Gibco. CpG 2006 and Cy5.5-labeled CpG 2006 were purchased from Genscript China. The stock solutions of un-labeled CpG in PBS were prepared as 100 μg/mL, while that of the labeled CpG was 70 μg/mL. Mouse TNF-α ELISA Kit (Biolegend), Mouse IL-6 ELISA Kit (Biolegend), Mouse MMP-3 ELISA Kit (Biolegend). LPS (Invitrogen), Lysotracker Red DNA-99 (Yeasen, China), DAPI (Invitrogen), Immunization grade bovine type II collagen solution (2 mg/mL, Chondrex), complete Freund’s Adjust (5 mg/mL, Chondrex), incomplete Freund’s Adjust (5 mg/mL, Chondrex), chloral hydrate (Energy, China), and isoflurane (RWD Life Science, China) were purchased from companies.

*Synthesis and characterization of PK-BPY*: Under nitrogen atmosphere, H-Lys-OMe 2HCl (5 g, 21.51 mmol), Boc-Lys(Boc)-OH (22.34 g, 64.50 mmol), EDCI (12.38 g, 64.50 mmol) and HOBt (8.71 g, 64.50 mmol) were dissolved in freshly distilled dichloromethane (CH_2_Cl_2_, 50 mL). The reaction solution was stirred with ice bath for 30~60 min followed by addition of DIPEA (35.60 mL, 215.10 mmol) and stirred at 25 °C for 1~2 days. Next, the reaction mixture was concentrated by evaporating CH_2_Cl_2_ under reduced pressure using a rotary evaporator, and then diluted with chloroform (CHCl_3_) to 120 mL. The resultant CHCl_3_ solution was washed three times with saturated NaHCO_3_, HCl (1 M) and saturated NaCl solutions, respectively. The organic phase was dried with anhydrous MgSO_4_ overnight, filtered to remove MgSO_4_, concentrated by removal of CHCl_3_ under reduced pressure, purified by column chromatography using CH_2_Cl_2_/methanol (1:10, v/v) as eluent, and dried under vacuum. The Boc protected G2 dendrimer was obtained. Then, G2 (3.64 g, 4 mmol) was treated with anhydrous CH_2_Cl_2_ (14.00 mL) and TFA (14.00 mL, 160 mmol) to stir overnight at 25 °C to put off Boc groups. Then the G2 dendrimer was obtained after the evaporation of CH_2_Cl_2_ and TFA under vacuum as a white powder, which was used in the next step without further purification. Next, Boc-Lys(Boc)-OH (23.82 g, 68.78 mmol), EDCI (13.18 g, 68.78 mmol) and HOBt (23.82 g, 68.78 mmol) were added to a solution of G2 dendrimer (10 g, 11.46 mmol) in freshly distilled CH_2_Cl_2_ (100 mL) under nitrogen atmosphere. The reaction mixture was stirred with ice bath for 30~60 min followed by addition of DIPEA (32.60 mL, 183.41 mmol) and stirred at 25 °C for 2~3 days. After repeating the purification procedures same as G2 dendrimer, a white solid of Boc protected G3 dendrimer was obtained. Finally, Boc protected G3 dendrimer was treated with NaOH solution (1 M) dissolved in methanol to activate demethylation. After stirring overnight at 25 °C, the methanol was removed by vacuum, then the mixture was diluted with CH_2_Cl_2_/distilled water (80/20 mL). Under vigorous stirring, HCl (1 M) was added to the solution dropwise to adjust the pH value to 3~4. Then the oil phase was dried with anhydrous MgSO_4_ overnight, filtered to remove MgSO_4_, and dried under vacuum to afford a white solid. Next, demethylated G3 dendrimer (4 g, 2.33 mmol), NH_2_-PEG2000-NH_2_ (1.86 g, 0.93 mmol), EDCI (0.45 g, 2.33 mmol) and HOBt (0.32 g, 2.33 mmol) were dissolved in freshly distilled CH_2_Cl_2_ (40 mL) under nitrogen atmosphere. The reaction mixture was stirred with ice bath for 30~60 min followed by adding the solution of DIPEA (0.82 mL, 4.66 mmol) in DMF (10 mL) dropwise, and the mixture was stirred overnight at 25 °C. The mixture solution was concentrated under vacuum, and the residue was precipitated with anhydrous diethyl ether overnight. A white solid of Boc protected PK was obtained after the removal of diethyl ether. Ultimately, PK conjugate was treated with TFA to expose amino groups according the steps mentioned before. PK-BPY was synthesized by specifically conjugating BPY to amino groups of PK. Briefly, under a nitrogen (N_2_) atmosphere, G3K (1.00 g, 0.12 mmol), BPY (0.048 g/0.24 mmol, 0.12 g/0.6 mmol, 0.24 g/1.2 mmol), HOBT (97 mg, 0.72 mmol), and HBTU (273 mg, 0.72 mmol) were first mixed in 5 mL of anhydrous DMF, and the mixture was stirred at 0 ℃ for 30 min, after which, DIPEA was added dropwise, and the mixture was stirred at room temperature (RT) for a further 24 h. The resulting PK-BPY was purified by sequential dialysis against DMF and distilled water for three days. Finally, PK-BPY was obtained after lyophilization.

*Preparation and characterization of CK nanogel:* DMF solution (10 mL) containing CsA (50 mg, 0.2 mmol) was added dropwise into the PK-BPY (500 mg, 0.06 mmol) in DMF solution (50 mL). The reaction mixture was stirred (1500 r min^−1^) at room temperature for 24 h, followed by extensive dialysis (Spectrum, MWCO 1000 Da) against DMF and H_2_O. Finally, CK was obtained after lyophilization. The contents of CsA loaded in PK-BPY were measured using ELISA. The DLC and DLE were calculated using the following equation: DLC (%) = (weight of CsA in CsA-loaded nanogels / weight of CsA-loaded nanogels) × 100%; DLE (%) = (weight of CsA in CsA-loaded nanogels / weight of CsA feeding) × 100%. Particle size and ζ-potential of nanogels were measured by dynamic light scattering (DLS, Nano ZS90). Morphologies of nanogels were observed by TEM (JEM-2100Plus).

*Preparation and characterization of CKS polyplexes:* To synthesize HA-SS-COOH, a solution of HA (100 mg) in 20 mL formamide was prepared, to which EDC (30 mg), NHS (20 mg), and DMAP (1.5 mg) were added. After stirring at room temperature for 2 hours to achieve complete dissolution, selenocystamine (50 mg) was added, and the mixture was stirred for an additional 12 hours. Post-reaction, the mixture was concentrated and precipitated in acetone to obtain a white precipitate (95.8 mg). This precipitate was then reacted with succinic anhydride (25 mg) and triethylamine (25 mg) in a fresh 20 mL formamide solution, stirred at 60 °C for 3 hours, and precipitated in acetone to obtain the desired HA-SS-COOH. Then, through electronic interaction, the anionic HA-SS-COOH was coated around the cationic CK nanogels and the resultant ternary polyplexes (CKS) was obtained.

*Cellular co-localization of CpG and CKS polyplexes:* 2 × 10^4^ cells/well of RAW264.7 cells were cultured overnight in a glass bottom dish. Then two experiments were conducted. The first one was to test if the CKS polyplexes could reduce the cell uptake of extracellular cfDNA. 1 μM of Cy5.5-labeled CpG 2006 with 1.0 μg/mL CKS polyplexes were added into the new culture media to replace the old media and incubated for 12 h. The second was to test whether CKS could bind intracellular cfDNA agonists and inhibit their stimulation to TLR9. After washing three times with fresh culture medium, 1 μM of Cy5.5-labeled CpG 2006 was added into the culture media and incubated for 4 h. The excessive CpG was removed by washing three times with PBS, then the CKS labeled with FITC in medium (1.0 μg/mL) were added. After 4, 8, and 12 h, the cells treated in these two experiments were stained with LysoTracker Red DNA-99 and DAPI for confocal microscopic observation (Leica SP8).

*Migration and invasion assays:* For assessment of cell migration, FLS were plated at a density of 10^5^ cells per chamber in serum-free DMEM in the upper chamber of transwell plates fitted with 8-mm pore membranes. DMEM supplemented with 10% FBS was added to the lower chamber as a chemoattractant. After 48 h, nonmigrating cells were removed from the upper surface and filters were stained with DAPI and phalloidin. For assessment of cell invasion, FLS were seeded at a density of 10^5^ cells per chamber onto Matrigel-coated transwell plates in serum-free DMEM. The lower chamber was filled with DMEM supplemented with 10% FBS. After 48 h, cells on the top surface were removed, and invaded cells were stained with DAPI and phalloidin.

*RT-PCR:* For RT-PCR quantification, RAFLS were seeded onto 6-well plates at density of 2 × 10^5^ cells per well for 48 h. Then, CKS (1.0 μg/mL) were added. After 48 h, the cells were harvested and total RNA was extracted using TRIzol reagent. The levels of mRNAs (including IL-6, CCL2, CCL20, MMP3, MMP13, IL10, TNFSF11, CCL5) were determined by RTPCR. GAPDH was used as a normalizing control. IL-6 primers were forward, 5’-GGAGCCCACCAAGAACGATA-3’ and reverse, 5’-ACCAGCATCAGTCCCAAGAA-3’, CCL2 primers were forward, 5’-AGGTCCCTGTCATGCTTCTG-3’ and reverse, 5’-TCTGGACCCATTCCTTCTTG-3’. CCL5 primers were forward, 5’-ACTCCCTGCTGCTITGCCTAC-3’ and reverse, 5’-GAGGTTCCTTCGAGTGACA-3’. CCL20 primers were forward, 5’-ACCATGTGCTGTACCAAG-3’ and reverse, 5’-ATGTCACAGCCTTCATTGG-3’. IL10 primers were forward, 5’-TGCAGGACTTTAAGGGTTACTTGG-3’ and reverse, 5’-GGCCTTGTAGACACCTTGGTC-3’. MMP3 primers were forward, 5’-GATGCCCACTTTGATGATGATGAA-3’ and reverse, 5’-AGTGTTGGCTGAGTGAAAGAGACC-3’. MMP13 primers were forward, 5’-CCCCAACCCTAAACATCCAAAAAC-3’ and reverse, 5’-TTAAAAACAGCTCCGCATCAACCT-3’. TNFSF11 primers were forward, 5’-AAACGCAGATTTGCAGGACTC-3’ and reverse, 5’-CCCCACAATGTGTTGCAGTTC-3’.

*Animal model induction and treatment:* All in vivo experiments were performed according to the Ethics Committee of Drum Tower Hospital and the approval number issued by the Laboratory Animal Welfare Ethics Committee of Drum Tower Hospital was 2021AE01008. At 42 days post-immunization, CIA DBA-1 mice were randomly assigned to six groups (n = 5 for each group) receiving intravenous injections of PBS, CsA, CK, CKH, CPS, and CKS every seven days until day 63. Hindpaws of treated mice were scored for RA severity using a scale of 0 to 4 for each paw every seven days. Hindpaws swelling was also measured with a plethysmometer under the same measurement frequency. After the mice were sacrificed, their hindlimbs were immediately collected and stored in paraformaldehyde (4%) at room temperature. The mouse hindlimbs were assessed by a micro-CT system (Sky-Scan, Bruker, Belgium). Scanning parameters were set at a voltage of 49 kV, a current of 179 μA, and an exposure time of 475 ms. The BMD of knee subchondral bone and anklebone were also measured from reconstructed 3D CT images using the software provided by Bruker Company. In addition, the collected limbs were used for histological analysis using H&E, toluidine blue, and safranin-O staining. The major organs were sectioned and stained by H&E. Staining was visualized using a digital microscope (Olympus, CX31, Japan). Blood samples from the mice were taken from the eye socket at day 63. The samples were centrifuged at 3000 rpm for 10 min after standing for 2 h. The supernatant was collected and recentrifuged at 3000 rpm for 10 minutes to obtain cell-free serum.

*Biodistribution of cationic materials:* At day 42 after immunization, the untreated CIA mice and normal mice were anesthetized (1.5–2% isoflurane, 0.5 ml/min oxygen), and the Alexa Fluor® 750 labeled CKS materials were intravenously injected. The biodistribution of the polyplexes was monitored by NIRF imaging using an in vivo imaging scanner (IVIS Lumina LT, Perkin Elmer) at different time points during 24 h. At 2 and 24 h, one from each group was sacrificed for dissection. The joints, heart, lung, liver, kidney, and spleen were taken out for ex vivo NIRF imaging, and their mean NIRF intensity was calculated by Bruker MI software.

*Statistical analysis:* A one-way analysis of variance (ANOVA) test via GraphPad software (Mac version) was used to analyze experimental data wherever appropriate.
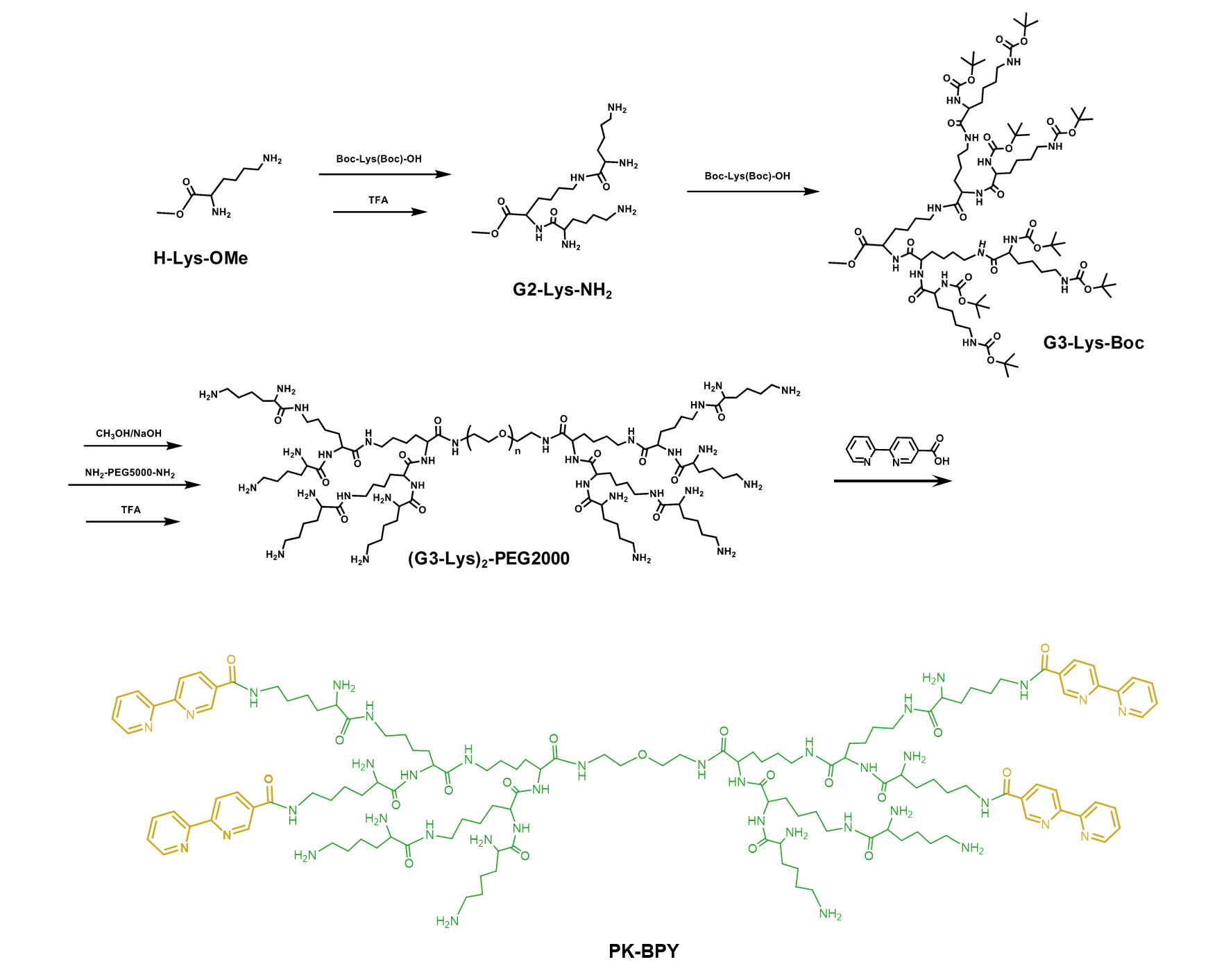


**Fig. S1.** Synthesis route of PK-BPY.

**
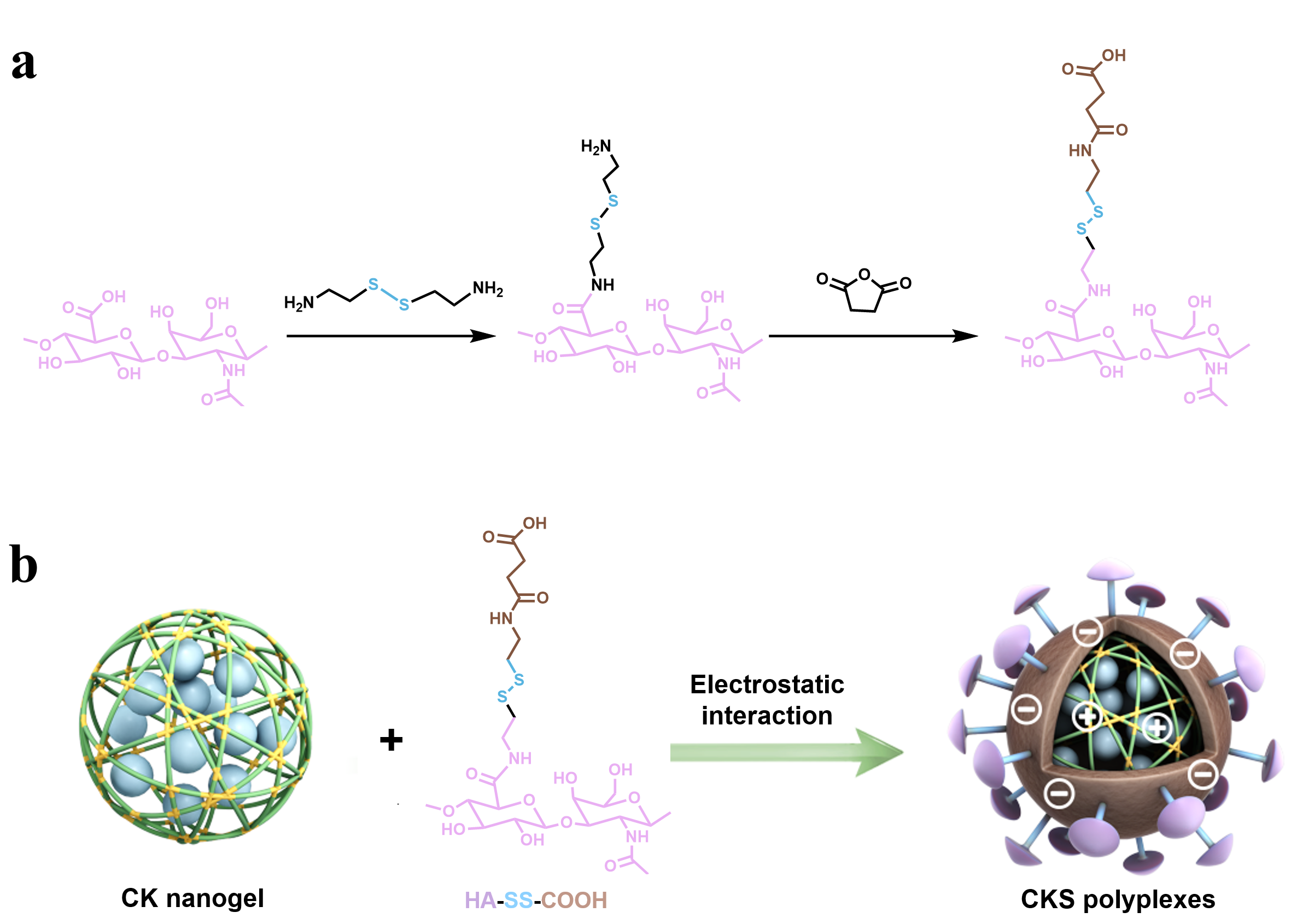
**

**Fig. S2.** ynthesis route of HA-SS-COOH and CKS polyplexes.

**Fig. S3.** MALDI-TOF spectra of G3K.

**Fig. S4.** ^1^H-NMR spectrum of a) PK (up), PK-BPY (down), b) HA (up), and HA-SS-COOH (down).


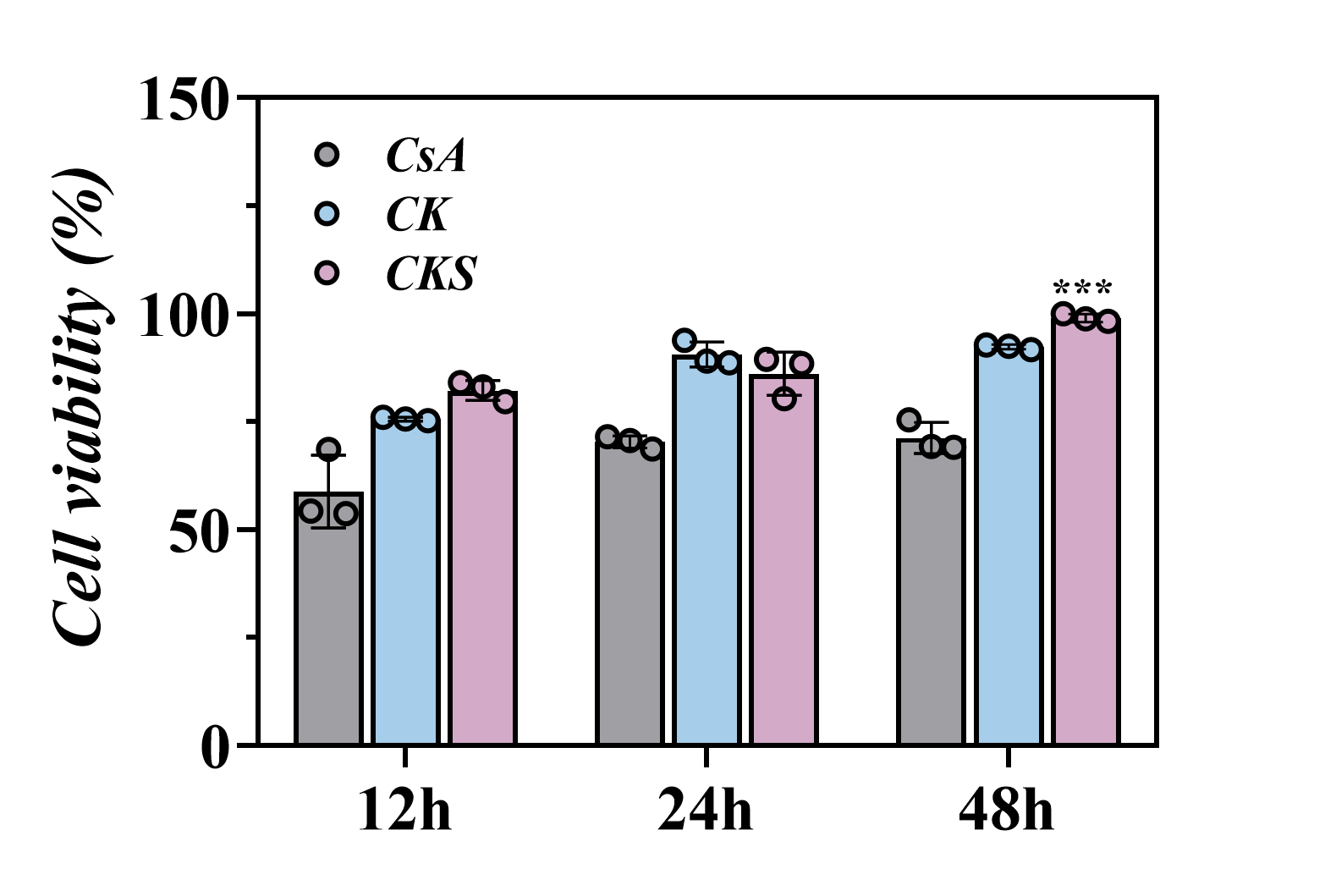


**Fig. S5.** Cell viability of CKS polyplexes.





**Fig. S6.** Synthesis route of FITC-labeled PKBPY.


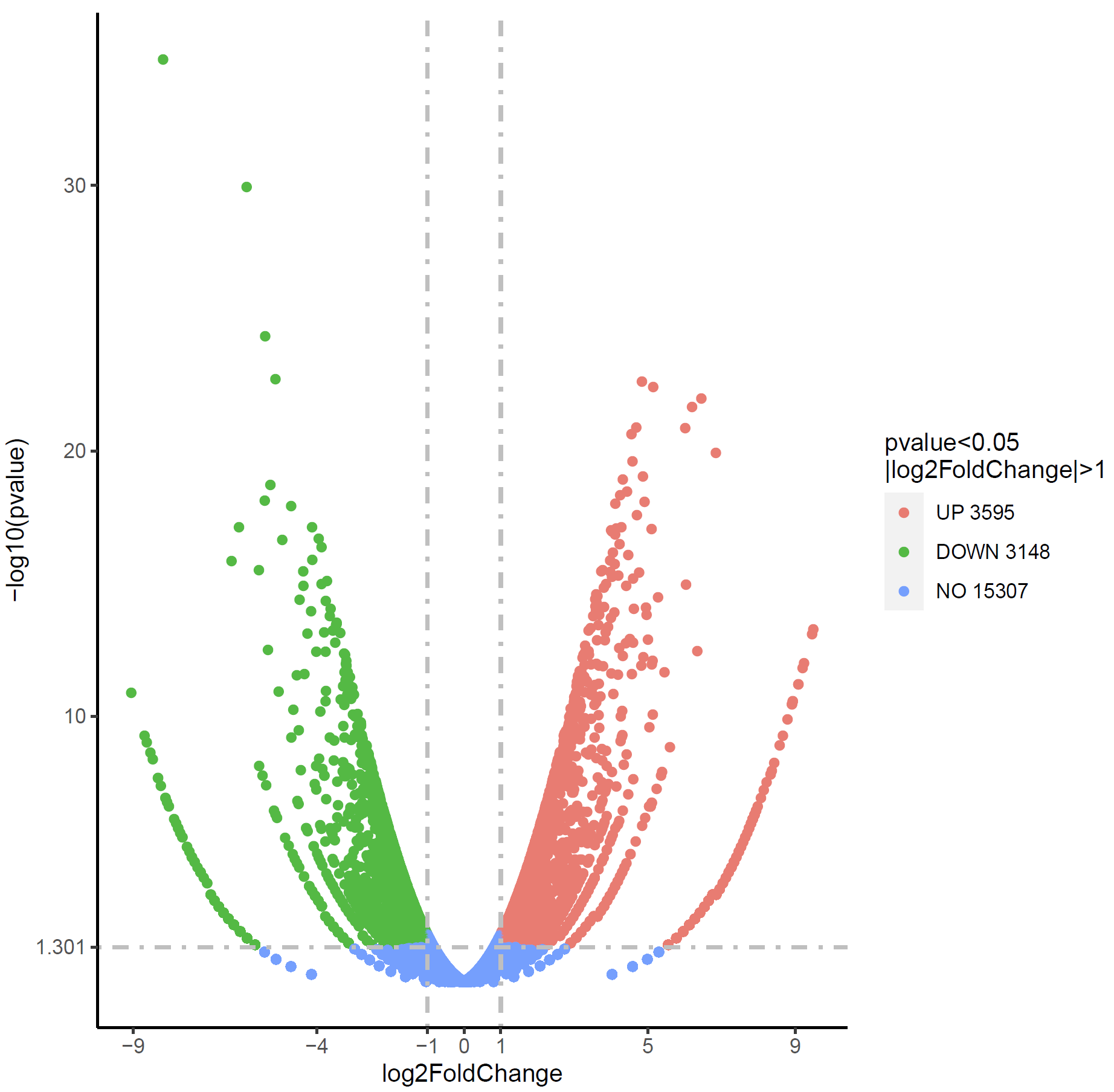


**Fig. S7.** Differential gene statistics. The abscissa was Log2 Fold Change value, the ordinate was -Log10 (*p value*), and the dashed blue line represented the threshold line of the differential gene screening criteria.


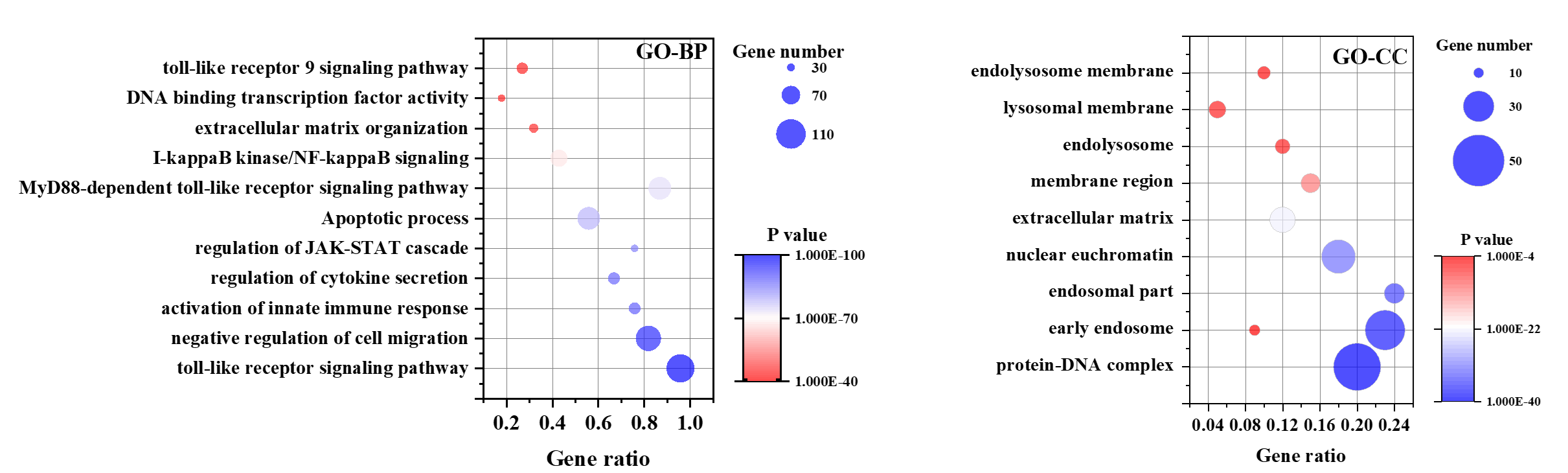


**Fig. S8.** GO analysis of top ten significantly expressed genes in CKS group compared with NC group. BP, biological processes; CC, cellular components**.**





**Fig. S9.** Synthesis route of AF750-labeled PKBPY.


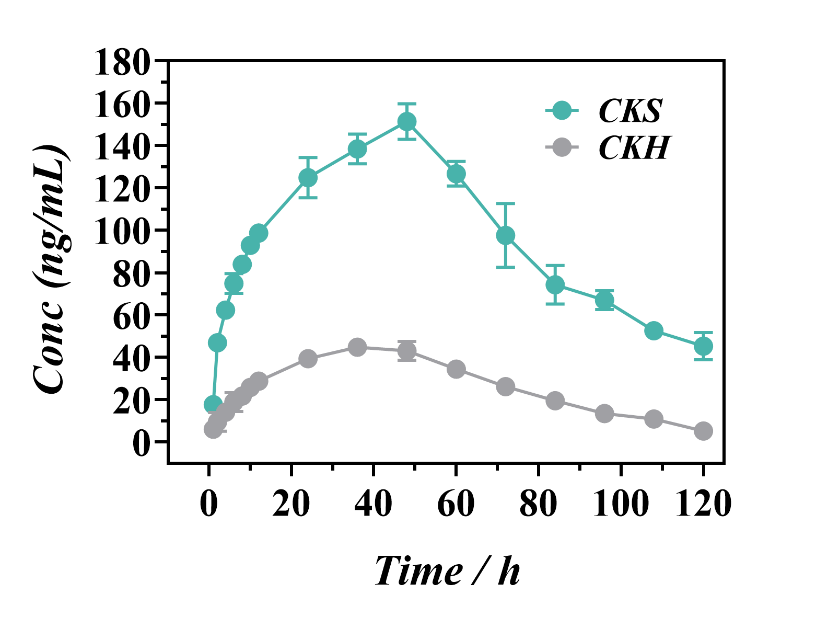


**Fig. S10.** Pharmacokinetics study of CKS and CKH.

**
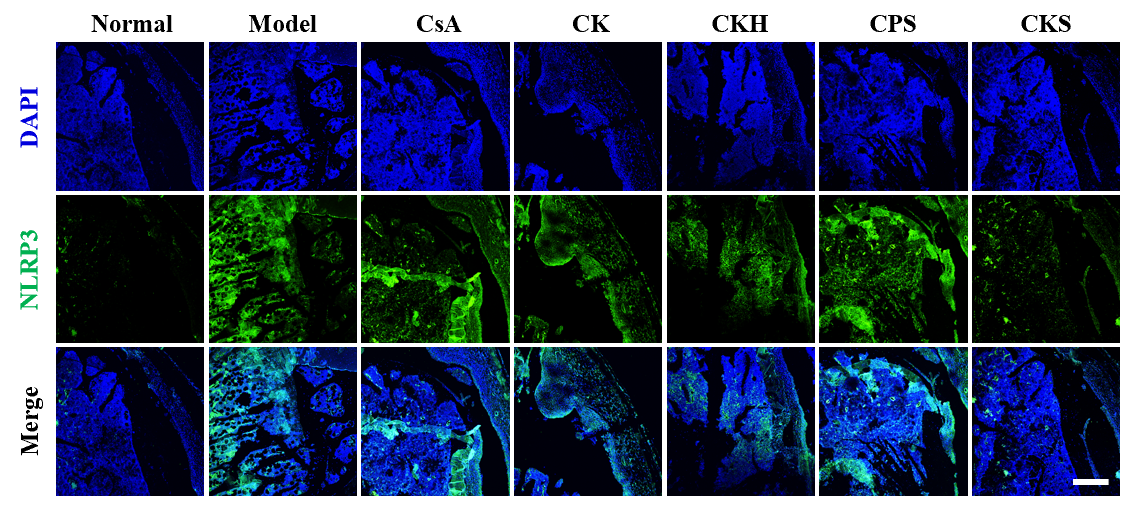
**

**Fig. S11.** Tissue sections were stained with DAPI and NLRP3. The scale bar is 200 μm.


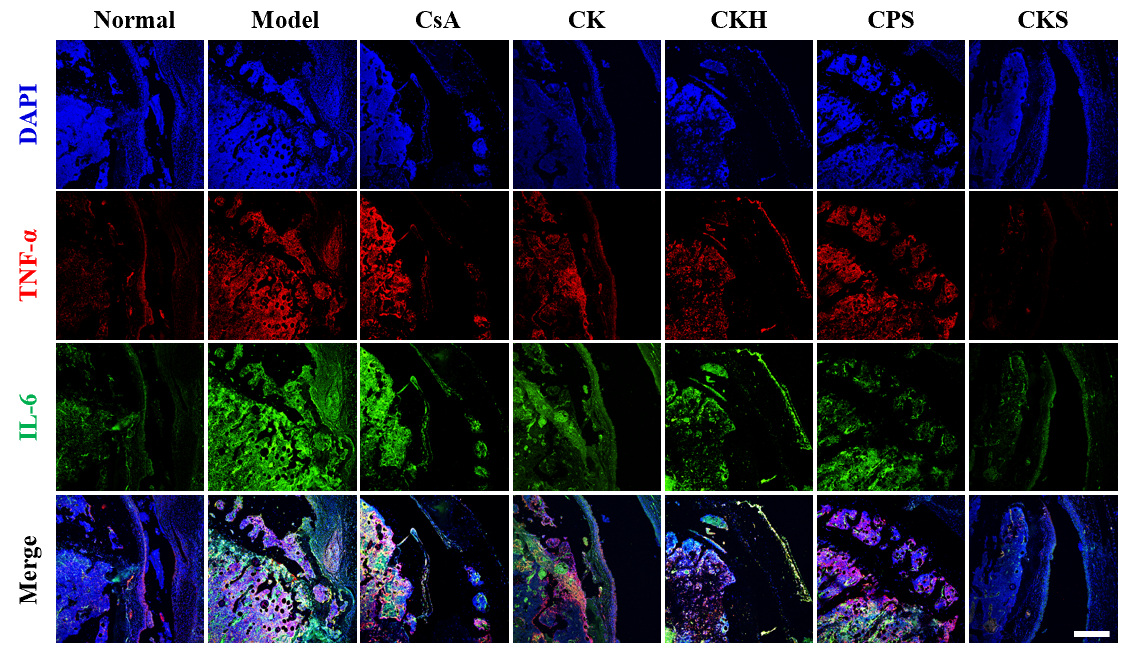


**Fig. S12.** Tissue sections were stained with DAPI, TNF-α, and IL-6. The scale bar is 200 μm.


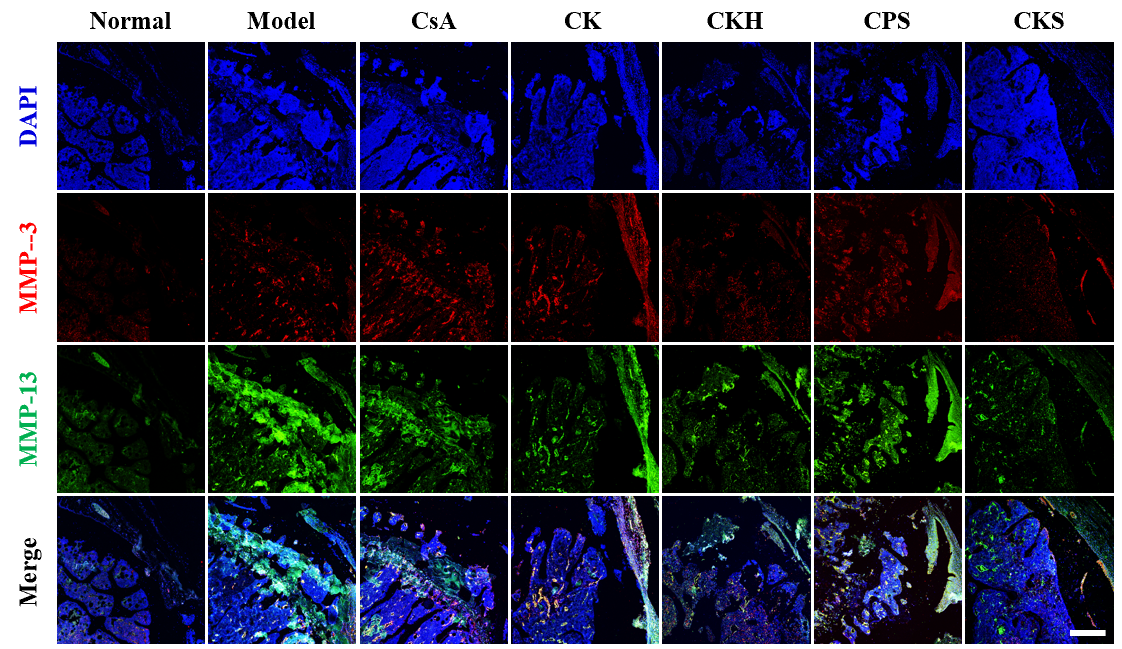


**Fig. S13.** Tissue sections were stained with DAPI, MMP-3, and MMP-13. The scale bar is 200 μm.

**
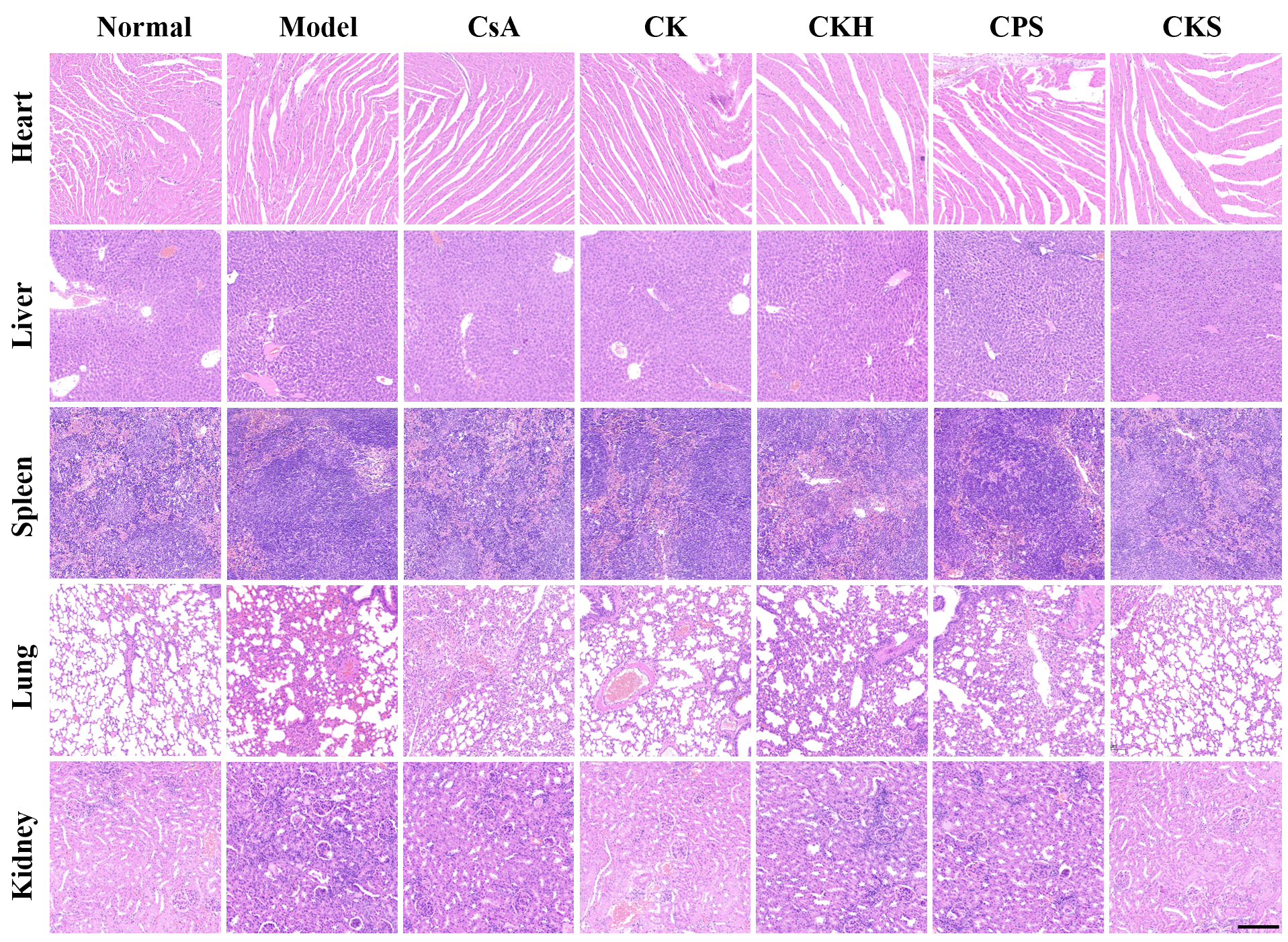
**

**Fig. S14.** H&E staining of heart, liver, spleen, and lung of mice. The scale bar is 200 μm.

**Movie S1 (separate file).** The inflamed joint homing ability of CKS polyplexes by injecting them into the tail veins of CIA mice.

**Movie S2 (separate file).** CKS polyplexes nanogels displayed minimal accumulation in the joints of normal mice
